# Effectiveness of a home telemonitoring program for patients with chronic obstructive pulmonary disease in Germany: Evidence from the first three years

**DOI:** 10.1371/journal.pone.0267952

**Published:** 2022-05-12

**Authors:** Florian Hofer, Jonas Schreyögg, Tom Stargardt

**Affiliations:** Hamburg Center for Health Economics (HCHE), Universität Hamburg, Hamburg, Germany; University of California San Francisco, UNITED STATES

## Abstract

**Introduction:**

Chronic obstructive pulmonary disease (COPD) affects more than 6 million people in Germany. Monitoring the vital parameters of COPD patients remotely through telemonitoring may help doctors and patients prevent and treat acute exacerbations of COPD, improving patients’ quality of life and saving costs for the statutory health insurance system.

**Objective:**

To evaluate the effects from October 2012 until December 2015 of a structured home telemonitoring program implemented by a statutory health insurer in Germany.

**Methods:**

We conducted a retrospective cohort study using administrative data. After building a balanced control group using Entropy Balancing, we calculated difference-in-difference estimators to account for time-invariant heterogeneity. We estimated differences in mortality rates using Cox regression and conducted subgroup and sensitivity analyses to check the robustness of the base case results. We observed each patient in the program for up to 3 years depending on his or her time of enrolment.

**Results:**

Among patients in the telemonitoring cohort, we observed significantly higher inpatient costs due to COPD (€524.2, p<0,05; €434.6, p<0.05) and outpatient costs (102.5, p<0.01; 78.8 p<0.05) during the first two years of the program. Additional cost categories were significantly increased during the first year of telemonitoring. We also observed a significantly higher number of drug prescriptions during all three years of the observation period (2.0500, p < 0.05; 0.7260, p < 0.05; 3.3170, p < 0.01) and a higher number of outpatient contacts during the first two years (0.945, p<0.01, 0.683, p<0.05). Furthermore, we found significantly improved survival rates for participants in the telemonitoring program (HR 0.68, p<0.001).

**Conclusion:**

On one hand, telemonitoring was associated with higher health care expenditures, especially in the first year of the program. For example, we were able to identify a statistically significant increase in inpatient costs due to COPD, outpatient contacts and drug prescriptions among individuals participating in the telemonitoring program. On the other hand, the telemonitoring program was accompanied by a survival benefit, which might be related to higher adherence rates, more intense treatment, or an improved understanding of COPD among these patients.

## Introduction

Telemonitoring is expected to enhance remote communication between patients and practitioners [1, 2] and thus to improve the provision of health care services, especially in rural areas [3]. A faster and easier way to communicate with health care providers has the potential to improve disease management substantially for older patients with chronic conditions such as chronic obstructive pulmonary disease (COPD). In the case of COPD, telemonitoring is expected to be a useful tool to initiate counter measures in a timely manner to prevent or contain acute exacerbations of a patient’s health status [4]. Indeed, intervening early might be an effective way to prevent patients from having to undergo intensive (and thus expensive) inpatient treatment. In Germany, approximately 13.2% of individuals above the age of 40 years have been diagnosed with COPD, totaling approximately 6.2 million people [5–7]. Together, the direct medical costs and indirect costs of COPD have been estimated at between €1,212 and €3,492 per patient per year [8].

Although the results of a number of studies suggest that telehealth interventions may be effective in other chronic conditions [9–14], evidence of their effectiveness among patients with COPD is inconclusive [15]. Recent systematic reviews of randomized controlled trials (RCTs) have found that the majority of published studies have reported that telehealth was effective at lowering heath care utilization in the short run [4, 15]. A substantial number of RCTs, however, have not found significantly lower costs among patients with access to telehealth [15]. Furthermore, the majority of RCTs conducted to date have had small sample sizes and study periods of short duration (i.e., only 2–12 months) [15–17]. When patients use new technologies, such as telemonitoring, they are expected to adjust their behavior slowly over time. This suggests that longer follow-up periods are needed to identify potential benefits [18]. However, even in observational studies with more than 12 months of follow up, the evidence has remained inconclusive. A recent observational study with 24 months of follow up found telemonitoring to be associated with lower rates of hospital admissions and a shorter average length of stay. The cohort consisted of only 191 patients and no risk adjustment was applied [19]. Another observational study, conducted by Steventon et al. (2016) in a predominantly rural area in England, followed 1432 patients with COPD, diabetes, or heart failure for up to 33 months. The authors found no reduction in secondary care utilization and an increase in emergency hospitalizations and outpatient attendance in the telemonitoring cohort. The authors used a 1:1 matching approach for risk adjustment in a time-to-event analysis. Approximately 65% of patients in the telemonitoring cohort had a diagnosis of COPD [20].

In addition to different methodological approaches [4, 21], other reasons for the inconclusive evidence on telemonitoring may be (a) the differing quality of standard health services for COPD patients in the country of interest [22], (b) a lack of clarity about which health measures or other variables can best predict acute exacerbations [23], and (c) differences in the target population (e.g., mean age and severity of COPD) [15–17]. The designs of the various telehealth interventions that have been studied to date have also been heterogeneous, differing mostly in (a) the number and types of vital parameters that have been transmitted (e.g., weight, pulse measurements, FEV_1_ values, blood oxygen saturation), (b) the nature of the contact between service providers and patients (e.g., telephone support vs. web video conferences), and (c) the content of the information provided (e.g., information about smoking cessation, exercise training) [15, 17].

In this study, we evaluated a home telemonitoring program offered in cooperation with the telemonitoring provider SHL Telemedizin by AOK Bayern, a large statutory health insurer in Germany providing coverage to approximately 4.4 million people. The chief aim of the program was to recognize exacerbations in COPD at an early stage and arrange timely countermeasures. This, in turn, was expected to prevent patients from being hospitalized, saving costs for the health insurer. The first year of the program was previously analyzed by Achelrod et al. They reported lower mortality rates in the telemonitoring cohort but no differences in health care related expenditures compared to the control group [24]. We were now able to investigate the effects of telemonitoring for up to three years in each patient, following 909 individuals who had access to telemonitoring in addition to standard care and comparing them to 6,917 individuals with access to standard care only.

## Methods

### Sample selection

We used a retrospective cohort study design to analyze patient-level data provided by AOK Bayern. We examined health care utilization and costs from the insurer perspective for patients receiving telemonitoring in addition to standard care compared to patients receiving standard care only. We analyzed data from 909 people with COPD who started to use telemonitoring equipment at some point between October 2012 and December 2015. Each person had been invited by letter from AOK Bayern to participate in the telemonitoring program. We used individuals insured by AOK Bayern but who did not participate in the telemonitoring program as a control group. All of the people in our sample were observable from the beginning of 2010 through the end of 2016 and had had a COPD-related inpatient stay within the 24 months (risk-adjustment period) before their individual index date.

For each person in the telemonitoring program, we defined the index date as the day that he or she started to use telemonitoring equipment. The median duration between the time when individuals were invited to participate in the telemonitoring program and the time they began to use the equipment was 99 days. We therefore defined the index date for the control group as 99 days after the initial invitation sent out by the health insurer.

We excluded individuals from either group if they (a) had not been continuously insured by AOK Bayern during the risk-adjustment and observation periods, (b) did not have a confirmed diagnosis of COPD, defined either as one COPD-related hospitalization or at least two COPD-related outpatient contacts within two consecutive quarters, (c) had been diagnosed with any of a predefined list of comorbidities (i.e., malignant neoplasms, cognitive disabilities, drug or alcohol addiction, severe heart failure, haemorrhagic diathesis, dialysis, or Parkinson’s or Alzheimer’s disease), or were receiving certain treatments (i.e., dialysis, long-term ventilation, chemo-/radiotherapy) that we assumed to might hamper participation in the telemonitoring program and thus limit its effectiveness a priori, (d) who were participating in other health care programs offered by AOK Bayern, (e) who had been rejected by the telemonitoring provider, SHL Telemedizin, or its practitioners (see S1 Table for more detailed information), (f) who had not had a COPD-related hospitalization in the two years before their individual index date, or (g) who had enrolled in the program after December 2015. We also excluded individuals if they were hospitalized at the time of their index date. Additionally, we excluded the 99-percentile with the highest amount of total costs within the two-year period before their individual index date, to avoid bias introduced by large outliers.

Compliance with ethical standards was approved by University of Hamburg. All insurees who participated in the telemonitoring pilot program have agreed to do so in writing. All data were fully anonymized before we accessed them.

### Study outcomes

Outcomes of interests were direct medical costs, health care utilization, and mortality over a period of three years. When measuring direct medical costs, we looked not only at total direct medical costs but also distinguished between the costs of (a) inpatient treatment, (b) outpatient treatment, (c) rehabilitation, and (d) pharmaceuticals. For health care utilization, we observed the number of (a) inpatient and outpatient contacts, (b) days spent in a hospital, and (c) drug prescriptions. Costs for telemonitoring services were unobservable due to the nature of the contract between SHL Telemedizin and AOK Bayern (i.e., a profit-sharing agreement) [24].

### Telemonitoring intervention

Patients were asked to use a spirometer twice a week to collect information regarding their lung function non-invasively which was transmitted automatically to a telemedicine center run by SHL. Patient data were monitored by doctors and nurses who could be contacted by telephone 24 hours a day, seven days a week. Patients were contacted by SHL whenever their lung function deteriorated below a threshold predefined by their pulmonologist. Countermeasures to prevent acute deterioration of health consisted either of additional information provided to patients on how best to manage their disease or the initiation of emergency management by SHL. All measures affecting the patient were communicated to the patient’s general practitioner or responsible specialist.

All patient information, such as that on vital parameters or contacts with SHL, was stored in an electronic patient record accessible by SHL, the patient, and the patient’s general practitioner or responsible specialist upon the patient’s agreement. The basic telemonitoring equipment consisted of a set top box and their spirometer. Additionally, an oximeter was provided instead of or in addition to the spirometer if forced expiratory volume in 1 second (FEV_1_) was below 35% at program start or fell below 35% afterwards (thus being available only to patients with GOLD status 3 or 4). Data measured via oximeter was also collected twice a week [25].

Lastly, patients were asked to fill in a questionnaire about their general well-being and complete a COPD assessment test (CAT) twice a week. At predefined intervals of two to three weeks, patients received educational content by telephone about smoking cessation and how to follow a healthier lifestyle [24].

### Statistical analysis

#### Health care utilization and costs

To account for confounding by observable and unobservable factors, we chose a two-step approach. In the first step, we performed entropy balancing to balance predefined covariates [26, 27] and account for confounding by observable factors [28]. Because the weights of individuals in the telemonitoring program were set to one while control units were assigned weights less than one, we estimated the average treatment effect on the treated (ATT) [29]. The set of predefined covariates was chosen based on the literature and consisted of socio-demographic data (i.e., age, gender, insurance contribution class, participation in a disease management program, GOLD status) and comorbidities. To account for comorbidities, we chose a combination of EIixhauser comorbidity groups based on ICD-10 codes [30, 31] and pharmacy-based metrics (PBM) based on the Anatomical Therapeutic Chemical (ATC) [32]. We collected all of the information necessary for the risk adjustment from the two years before each patient’s index date, which could fall between October 2012 and December 2015.

In the second step, we estimated three difference-in-differences (DiD) models, one for each program year. Our model specification to identify the intention-to-treat effect of telemonitoring was as follows:

$$Y_{it}=\beta_{0}+\beta_{1}{Treat}_{it}+\beta_{2}{After}_{it}+\beta_{3}{Treat*After}_{it}+\varepsilon_{it}$$ (1)

where Y_it_ are the outcomes of interest for individual *i* in period *t*. The DiD estimator of interest is *β*_*3*_, which indicates the average difference in outcomes due to the telemonitoring program in the intervention periods. Robust standard errors were used to account for heteroscedasticity. To examine whether the key assumption of parallel trends of individual attributes holds, we performed placebo DiD regressions by defining the year two years before the intervention period as the pre-period and the year before the intervention period as the post period. None of the DiD estimators reached statistical significance for any outcome, indicating that the parallel trends assumption holds.

#### Mortality

As a baseline model, we estimated a Cox proportional hazards model and chose all variables previously used for risk-adjustment as covariates. The inclusion of all covariates instead of weights resulting from balancing/matching has been reported previously as a valid method for large cohorts [33]. Nevertheless, to check the robustness of our estimates, we also estimated a Cox regression model using weights obtained from entropy balancing. Time to event (i.e., whether a patient died) was measured in days starting at the individual’s index date. The outcome of interest was all-cause mortality.

### Subgroup analysis

We performed subgroup analyses by distinguishing among COPD patients according to their GOLD status. By separately analyzing patients with an (a) GOLD status of 1 or 2, (b) GOLD status of 3, or (c) GOLD status of 4, we were able to assess whether patients with a higher ex-ante risk of exacerbations (i.e., individuals with a higher GOLD status) benefitted more or less from telemonitoring than their lower-risk peers [15, 34]. Individuals in the control group were again assigned weights obtained from entropy balancing. Because creating subgroups resulted in samples of substantially smaller size compared to the base case analysis, some covariates had to be dropped for the entropy balancing step due to collinearity.

### Sensitivity analysis

To check the robustness of our results, we (a) excluded deceased patients from our base case analysis and (b) included the 99-percentile of patients with the highest costs in the two-year period prior to the individual index dates.

To investigate whether potential differences in survival were biased by non-observable factors, we performed two additional survival analyses. We used information about individuals who were willing to participate in the telemonitoring program but could not be included for various reasons (see S1 Table). We shall call these patients “interested non-participants” in the rest of this paper. Our idea was that interested non-participants would be more comparable to actual participants in terms of unobservable factors, such as motivation. We therefore compared survival in interested non-participants (as long as they would have met the inclusion criteria for the telemonitoring group of our base case analysis) to that in participants in the telemonitoring group and individuals in the control group. Patients who started to use telemonitoring equipment but were excluded from the study shortly thereafter (N = 12) were still excluded from our analyses and do not belong to the group of interested non-participants.

## Results

### Descriptives and balancing

**Fig 1:**
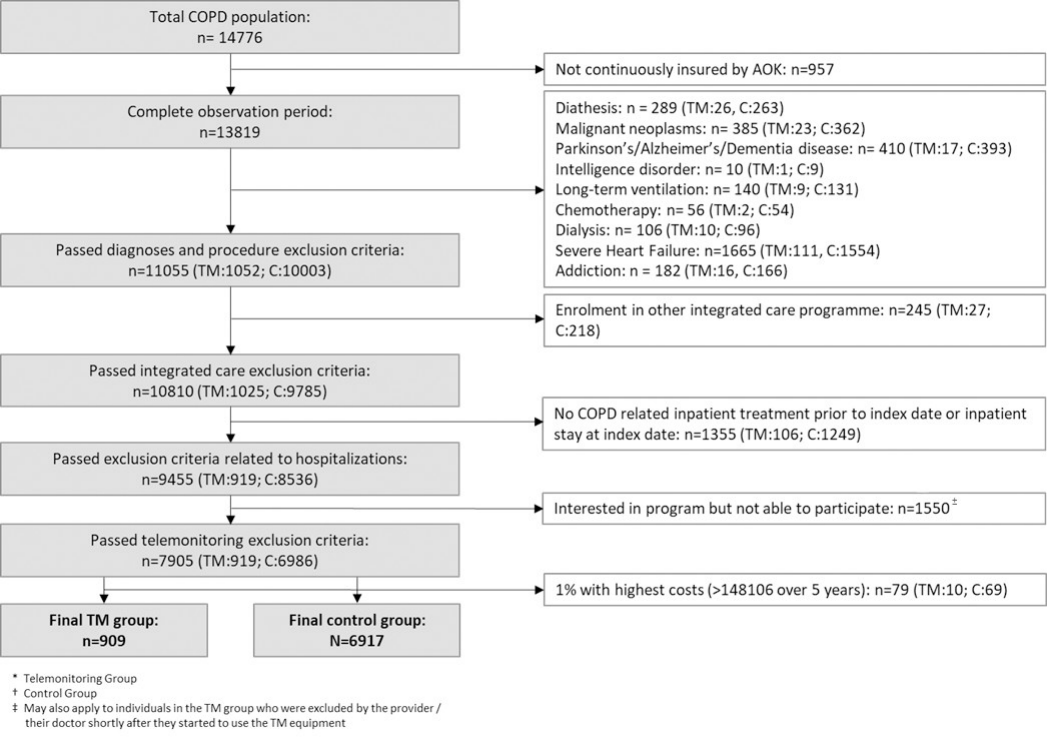
Selection criteria. TM = telemonitoring group, C = control group, ± may also apply to individuals in the telemonitoring group who were excluded by the provider or their physician shortly after telemonitoring started.

The original data set contained information on 14,781 individuals. Applying our inclusion criteria yielded a final sample of 7,826 patients with COPD, of whom 909 used telemonitoring in addition to standard care and 6,917 of whom received standard care only (Fig 1). Before balancing, the standardized mean differences of 19 covariates exceeded the target threshold of 10%. The cohorts differed substantially in age, insurance contribution class, and participation in disease management programs. After balancing, no standardized difference exceeded 10%, indicating a good balance of covariates. More detailed information on baseline characteristics before and after balancing is given in Table 1 and S2 Table.

**Table 1:** Baseline characteristics of telemonitoring (TM) and control group before and after entropy balancing (EB).

Variables	TM	Control		SMD	
		before EB	after EB	before EB	after EB
*Sample size (N)*	*909*	*6*,*917*			
Mean age (years)	63.85	67.61	63.84	-0.39	0.00
Male	55.23	50.21	55.21	0.10	0.00
FEV_1_ values:					
FEV_1_≥70%	7.59	9.70	7.59	-0.08	0.00
70%>FEV_1_≥50%	16.83	19.78	16.83	-0.08	0.00
50%>FEV_1_≥35%	26.07	20.46	26.07	0.13	0.00
FEV_1_<35%	35.31	25.43	35.31	0.21	0.00
FEV unknown	14.20	22.63	14.20		
Tobacco addiction	40.48	28.84	40.48	0.24	0.00
Insurance status:					
mandatory	33.22	27.70	33.22	0.12	0.00
pensionary	60.07	66.23	63.05	-0.13	0.00
voluntary	6.71	6.07	6.73		
DMP enrolment:					
DMP COPD	44.99	27.74	44.99	0.35	0.00
DMP Asthma	8.80	7.71	8.80	0.04	0.00
DMP Chronic Heart Failure	8.91	9.70	8.91	-0.03	0.00
DMP Diabetes Type 2	15.84	15.25	15.84	0.02	0.00
Elixhauser comorbidities (see S2 Table)					
before EB	4 of 31 with SMD > 0.1				
after EB	0 of 31 with SMD > 0.1				
Pharmacy-based classes (see S2 Table)					
before EB	5 of 31 with SMD > 0.1				
after EB	0 of 31 with SMD > 0.1				

All values in % unless indicated otherwise

SMD = Standardized mean difference

FEV_1_ = Forced expiratory volume in 1 second

DMP = Disease management program.

### Base case analysis

The results of our base case analysis after balancing are given in Table 2. Following the ITT approach, we observed statistically significant differences in costs between the telemonitoring and standard care cohorts foremost in the first year of the analysis. Within this first year, inpatient costs (COPD and non-COPD related), outpatient costs and total costs were increased in the telemonitoring cohort. In the second year, we only observed differences in inpatient costs due to COPD and outpatient costs. Virtually no differences were observed during the third year of the program.

**Table 2:** Base case regression results. Telemonitoring compared to standard of care.

	Year 1	Year 2	Year 3
*Cost outcomes*			
Inpatient costs	785.4*	331.8	302.9
Inpatient costs due to COPD	524.2*	434.6*	-31.1
Outpatient costs	102.5**	78.8*	-16.2
Cost for pharmaceuticals	141.6	300.4	120.3
Cost for rehabilitation	19.9	-7.0	16.7
Total costs	1049.4**	704.0	423.7
*Resource consumption*			
Inpatient contacts	0.141*	0.130	-0.042
Days in hospital	0.640	0.708	-0.331
Inpatient contacts due to COPD	0.080*	0.078	-0.004
Days in hospital (due to COPD)	0.711	0.758	-0.217
Inpatient contacts (emergency)	0.087*	0.074	-0.036
Days in hospital (emergency)	0.596	0.400	-0.189
Outpatient contacts	0.945**	0.683*	0.250
Drug prescriptions	2.118*	2.162*	2.560*

* < 0.05

** < 0.01

*** < 0.001.

We observed a slightly but significantly higher number of all-cause hospitalizations (0.141, p<0.05), COPD-specific hospitalizations (0.080, p<0.05) and emergency hospitalizations (0.087, p<0.05) in the telemonitoring group in the first year. Furthermore, we observed a significantly higher number of outpatient contacts in the telemonitoring group in the first two years of observation (0.945, p<0.01; 0.683, p<0.05). The number of drug prescriptions was significantly higher in the telemonitoring group in each of the three years (2.118, p<0.05; 2.162, p<0.05; 2.560, p<0.05).

**Fig 2:**
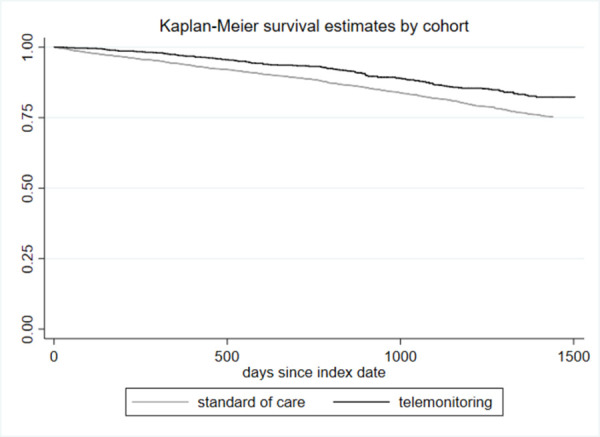
Kaplan-Meier survival estimates.

Telemonitoring was associated with longer survival in the baseline Cox model (HR 0.65, p<0.001). The survival benefit was still statistically significant but smaller after adjusting for bias due to observables through entropy balancing (HR 0.69, p<0.001). Unadjusted Kaplan-Meier curves are shown in Fig 2. Schoenfeld’s residual test indicated that the proportional hazards assumption holds for both the full regression model (p = 0.4866) and the model with EB weights (p = 0.1077).

### Subgroup analysis

The sample sizes of subgroups were substantially smaller compared to the base case analysis. Divided into (a) GOLD 1 or 2, (b) GOLD 3, and (c) GOLD 4, the telemonitoring group consisted of 222, 212, and 321 individuals, respectively. In all subgroups, the majority of differences in outcomes did not reach statistical significance which might be associated with the smaller sample sizes. Individuals with GOLD status 1 or 2 still showed a higher number of inpatient contact due to COPD and outpatient contacts in the first year of observation. In contrast to the base case analysis, this subgroup showed significantly reduced inpatient and total costs in the second year of the program while GOLD 4 patients showed higher expenditures in these categories in the first two years of observation. Also in contrast to the base case analysis, the number of drug prescriptions was not significantly higher in any of the subgroups. For more detailed information see Table 3.

**Table 3:** Subgroup analysis per GOLD status.

	GOLD 1 & 2 (N = 222/2,043)			GOLD 3 (N = 212/1,415)			GOLD 4 (N = 321/1,759)		
	Year 1	Year 2	Year 3	Year 1	Year 2	Year 3	Year 1	Year 2	Year 3
*Cost outcomes*									
Inpatient costs	-441.5	-1,122.9*	-191.0	1,128.7	-174.0	49.0	1,174.6	1,221.0	-299.5
Inpatient costs due to COPD	306.2	-93.5	-288.0	325.0	322.9	35.5	764.0*	1,004.8*	-270.5
Outpatient costs	146.6	54.73	-36.0	97.67	55.0	25.6	51.1	114.0	-40.53
Cost for pharmaceuticals	-73.8	-191.0	32.4	175.2	70.41	175.3	207.9	830.4*	154.4
Cost for rehabilitation	-62.2	-56.0	-136.3	41.83	-138.9*	131.9	35.88	63.3	-7.87
Total costs	-430.8	-1.315.2*	-330.9	1,443.4*	-187.5	381.7	1,469.5*	2,228.6*	-193.4
*Resource consumption*									
Inpatient contacts	0.014	0.012	-0.137	0.335**	0.048	-0.073	0.103	0.242	-0.240
Days in hospital	-1.126	-1.311	-0.464	1.926	-0.667	-2.044	0.247	1.744	-2.868
Inpatient contacts due to COPD	0.084*	0.026	0.013	0.143	0.052	0.035	0.054	0.145	-0.139
Days in hospital (due to COPD)	0.484	-0.281	-0.407	1.113	0.267	-0.287	0.494	1.854	-1.431
Inpatient contacts (emergency)	0.077	-0.002	-0.037	0.187*	0.136	-0.048	0.038	0.040	-0.185
Days in hospital (emergency)	-0.244	-0.687	-0.548	1.174	0.602	-1.423	0.320	0.137	-1.345
Outpatient contacts	0.669**	0.678	-0.555	1.187*	0.904	0.673	0.680	0.615	0.323
Drug prescriptions	2.036	1.354	1.617	2.745	3.308	2.832	1.557	2.290	1.152

* < 0.05

** < 0.01

*** < 0.001.

### Sensitivity analysis

Excluding deceased patients from both groups did not alter the results substantially. However, after doing so, we found no more differences in the number of drug prescriptions.

Similarly, including the 99-percentile of patient with the highest health care expenditures prior to their index date changed the results only minorly. Please see Table 4 for more detailed information.

**Table 4:** Sensitivity analysis II: Excluding deceased / full sample.

	Deceased excluded			Full sample		
	Year 1	Year 2	Year 3	Year 1	Year 2	Year 3
*Cost outcomes*						
Inpatient costs	612.8*	376.1	436.3	603.8	-366.4	351.8
Inpatient costs due to COPD	536.3**	416.9*	50.8	587.9*	153.1	-159.5
Outpatient costs	103.7**	62.6	-1.7	99.3**	85.8*	-21.0
Cost for pharmaceuticals	120.7	291.9	102.2	237.9	425.2	294.9
Cost for rehabilitation	8.9	-15.1	18.1	12.1	-20.8	-1.7
Total costs	846.1*	715.5	554.9	953.0*	123.8	623.9
*Resource consumption*						
Inpatient contacts	0.138*	0.114	-0.034	0.126*	0.100	-0.036
Days in hospital	0.442	0.721	-0.072	0.405	-0.021	-0.428
Inpatient contacts due to COPD	0.083*	0.067	-0.004	0.076*	0.073	-0.002
Days in hospital (due to COPD)	0.764	0.756	-0.240	0.737	0.483	-0.256
Inpatient contacts (emergency)	0.088*	0.066	-0.023	0.078	0.058	-0.032
Days in hospital (emergency)	0.602	0.469	-0.057	0.483	0.111	-0.108
Outpatient contacts	0.871**	0.592	0.286	0.944**	0.707*	0.222
Drug prescriptions	1.647	2.031	2.206	2.126*	2.244*	2.439

* < 0.05

** < 0.01

*** < 0.001.

When comparing survival among participants in the telemonitoring program with that among interested non-participants, we observed a smaller hazard ratio for participants in the weighted regression model (HR 0.71, p < 0.01). For the full regression model the difference in the hazard ratio was not statistically significant (HR 0.77, p < 0.119). In contrast, we observed smaller hazard ratios for both the full regression model (HR 0.82, p < 0.01) and the weighted regression model (HR 0.82, p < 0.01) when we compared the survival of patients in our control group to that of interested non-participants.

## Discussion

We compared individuals with COPD who received standard care plus telemonitoring to those who received standard care alone. The main feature of the telemonitoring intervention was the automated transmission of data on vital parameters to a telemedicine center. Health professionals at the telemedicine center informed patients and their doctors if vital parameters exceeded individualized, predefined thresholds. Additionally, patients were provided with educational content on a regular basis. The rationale behind the telemonitoring intervention was to recognize acute exacerbations early in order to prevent COPD-related hospitalizations.

In our analysis, expenditure for inpatient treatment and the number of hospitalizations were increased during the first year of the program, while COPD related inpatient costs were increased in the first two years. We did not observe any reduction of inpatient related outcomes. In this regard, our results are comparable with those of several previously conducted RCTs [22, 35–37] and an observational study from the UK [20]. The results are also in line with those previously reported by Achelrod et al. who evaluated the first year of the telemonitoring program with smaller cohorts [24]. In contrast, two systematic reviews reported that the majority of RCTs found telemonitoring to be effective in reducing hospital admissions [4, 15]. The authors of one of these systematic reviews, however, pointed out that the overall quality of the included RCTs was poor [4].

There are two plausible explanations why our results (and those of other studies that show no effects of telemonitoring) differ from those of several previous studies. First, whether telemonitoring shows incremental effects compared to standard care probably varies depending on the quality of standard care in any given country [4, 22]. In countries or communities with very high quality standard care, telemonitoring may not provide any appreciable benefits. Second, our results might also be explained by difficulties in predicting exacerbations using information derived from the telemonitoring program. In the evaluated telemonitoring program, alerts were triggered according to a standardized protocol, i.e., when (a) patients exceeded 28 points in the CAT questionnaire, (b) their FEV_1_ value deteriorated by more than 20% or (c) the blood oxygen saturation of patients with severe COPD fell below 88%. However, Burton et al. reported that variations in vital parameters among patients before an exacerbation were quite high, making it difficult to predict acute health deteriorations in COPD patients reliably [38]. Additionally, Sanchez-Morillo et al. argued that the prognostic value of telemonitoring systems for COPD patients has been low due to a lack of a common definition for exacerbations, as well as to underreporting, which together may result in there being too little data for successful predictions [39]. Also, there is little evidence on which patient-related parameters, whether alone or in combination, are especially valuable for predicting exacerbations of COPD. One parameter that has been found to be potentially useful for such predictions in the past is blood oxygen saturation [23]. In the telemonitoring program evaluated in our study, blood oxygen saturation was measured and transmitted only by patients with severe COPD. However, we found no evidence of cost savings or reduced resource consumption in this subgroup of patients. That being said, we had no information on whether these individuals actually performed such measurements.

We observed a significantly higher number of outpatient contacts and costs and drug prescriptions among participants in the telemonitoring program. One possible explanation for this is that participation in the telemonitoring program may have improved doctors’ adherence to treatment guidelines, disease awareness among patients, or both. Similar results have been reported by studies evaluating German disease management programs for COPD [40, 41]. Nevertheless, the higher number of outpatient contacts and drug prescriptions among individuals in the telemonitoring program does not appear to be related to earlier detection of exacerbations. We did observe almost no differences in COPD-related hospitalizations (except for a slight increase in the first year of the program) or the number of days an individual spent in a hospital.

There also seems to be a tendency for TM to result in less costs for GOLD 1 & 2 patients, while resulting in higher costs for Gold 3 & 4 patients. However, our study lacks the power for a definitive conclusion on these subgroups. In particular, as GOLD 1 & 2 patients were not the primary focus of the analysed TM program and are underrepresented compared to their share in all COPD patients.

One of our most important findings is that of improved survival rates in the telemonitoring program, which were also previously reported by Achelrod et al. although to a smaller extend [24]. As our results also show a higher number of outpatient contacts and drug prescriptions, better survival might be related to improved adherence or understanding of the underlying disease among the participants. To investigate whether our results might be biased due to unobservable factors affecting the choice to participate in the program, we compared survival among individuals in our intervention and control groups with survival among interested non-participants who would have met the inclusion criteria for our intervention group. The differences in survival between participants and interested non-participants were smaller than those between participants and individuals in our control group and statistically significant in the weighted regression model. However, we also found differences in survival between interested non-participants and individuals in our control group. These finding suggest that at least part of the survival benefit can be attributed to unobservable factors that we were unable to control for. Thus, we overestimate the true effect of the telemonitoring program. Still, since the effects were (a) stronger in the telemonitoring group and (b) also present when comparing telemonitoring participants to interests non-participants it is not likely that the estimated differences are due solely to unobserved characteristics. However, as we also observed a higher utilization of health care services in the telemonitoring group, it is possible that an improvement in overall survival could also be achieved through closer patient care without additional telemonitoring. Nevertheless, it is important to note that the higher utilization of health services in this study were probably triggered by the telemedical monitoring. This being said, our results differ from those of an earlier observational study that used 1:1 matching and found no difference in mortality rates between a telemonitoring and a control group after up to 33 months [20]. One explanation may be that this earlier study had a very different setting, namely a predominantly rural area in England, and also included patients with diabetes or chronic heart failure.

The use of machine learning algorithms would probably add further value to the telemonitoring system evaluated in this study and might help it achieve its expected potential to reduce health care utilization [22, 39]. However, training these algorithms effectively would probably require more frequent data transmission [39, 42], which in turn could have a negative impact on compliance and even increase the number of dropouts [16]. Although patients had to transmit data to SHL only twice a week, approximately 11% of patients dropped out of the telemonitoring program during the course of the intervention. Unfortunately, we were unable to obtain information about the reasons for these dropouts.

The results presented in our study should be interpreted with care due to several important limitations. First, as this study is an observational study, we cannot conclude on a causal effect between telemonitoring and our outcome parameters. Although we accounted for this by applying entropy balancing and using a difference-in-differences approach. Furthermore, the intervention consisted of two components, namely educational content to enhance self-management and the monitoring of vital signs. Since the impact of these two components could not be separated, we were not able to determine which of them was the main driver of our results [19, 43]. Second, we did not have any information on the actual costs of the telemonitoring services due to the nature of the contract between AOK Bayern and SHL (i.e., a profit-sharing agreement). Including these costs would increase direct medical costs in the telemonitoring cohort which were already increased by an increase in health care provided. Third, 2,191 individuals who were willing to participate in the telemonitoring program could ultimately not do so because of various reasons and were subsequently excluded from our base case analysis. Of these individuals, 1,665 would have met the inclusion criteria for this analysis. Although our difference-in-difference approach accounts for unobservable time-independent factors, the exclusion of these individuals might still have induced bias, especially because some of them were not permitted to participate by the telemonitoring provider, their statutory health insurer, or their doctor. Thus, our results, particularly those of our survival analysis, should be interpreted with caution. Fourth, because we used administrative data (i.e., secondary data) provided by a large statutory health insurer in Germany, we were not able to analyze any clinical information that might have added substantial value to our study. Additionally, we were not able to monitor the compliance of individuals in the telemonitoring cohort. Fifth, GOLD status and thus disease severity was unknown for 21% of individuals in our sample because it was not coded, limiting our ability among to distinguish between individuals with mild, moderate, or severe COPD.

## Conclusion

To our knowledge, this is one of the largest observational studies to date to evaluate telemonitoring support and its effects on health-related outcomes among COPD patients for a period of up to three years while comparing them to a matched control group. Whereas we found no differences in costs, we did find a significantly higher number of outpatient contacts and drug prescriptions in the telemonitoring group. There seemed to be a tendency for costs with TM to be lower for GOLD 1 & 2 patients while being higher for GOLD 3 & 4 patients. We also found improved survival rates for individuals in this group. Although our study design does not allow for definitive conclusions regarding causal effects, our finding are in line with previously published studies which examine the effects of telemonitoring in COPD patients. Thus, our study contributes to a growing body of evidence suggesting that telemonitoring in its current form may not be able to prevent acute exacerbations in COPD reliably and may therefore be unable to save inpatient costs over the short term, at least in countries where standard COPD treatment is of high quality. Yet we are also one of the first studies to suggest that telemonitoring is associated with a long-term survival benefit. While we were unable to determine the underlying process leading to this benefit, possible explanations may be enhanced adherence, more intense treatment, or an improved understanding among patients of their underlying disease.

## Supporting information

S1 Table Reasons for refusal of telemonitoring services. (DOCX)

S2 Table Detailed information about differences in comorbidity between cohorts. (DOCX)
