# Supplementary material for: Effectiveness of a home telemonitoring program for patients with chronic obstructive pulmonary disease in Germany: Evidence from the first three years
Source: PLoS One. 2022 May 12;17(5):e0267952. doi: 10.1371/journal.pone.0267952 (PMC9098037; doi:10.1371/journal.pone.0267952)
Supplement: S1 Table — (DOCX) [file pone.0267952.s001.docx]

**S1 Table. Reasons for refusal of telemonitoring services**

| **Reason** | **Frequency** |
| --- | --- |
| Refused by patient | 5 |
| Other reasons | 458 |
| Patient prioritizes other disease | 77 |
| Patient states that current degree of care suffices | 120 |
| Refused by sickness fund | 73 |
| Patient did not meet inclusion criteria | 552 |
| Patient feels healthy | 36 |
| Patient was not adherent | 8 |
| Patient is overstrained | 33 |
| Patient did not respond after initial expression of interest | 55 |
| Service refused by SHL | 4 |
| Service refused by practitioner | 216 |
| Patient deceased prior to service start | 7 |
| Patient is too often away from home for a long time | 21 |
| **Total** | **1665** |
