# Supplementary material for: Effectiveness of a home telemonitoring program for patients with chronic obstructive pulmonary disease in Germany: Evidence from the first three years
Source: PLoS One. 2022 May 12;17(5):e0267952. doi: 10.1371/journal.pone.0267952 (PMC9098037; doi:10.1371/journal.pone.0267952)
Supplement: S2 Table — (DOCX) [file pone.0267952.s002.docx]

**S2 Table. Detailed information about differences in comorbidity between cohorts**

|  |  | **TM (in %)** |  | **Control (in %)** | | | |  | **SMD** | | | | |  |
| --- | --- | --- | --- | --- | --- | --- | --- | --- | --- | --- | --- | --- | --- | --- |
|  |  |  |  | **pre** | | **post** | |  | **pre** | | | **post** | |  |
| **Elixhauser comorbidity groups** | |  |  | |  |  | |  |  | | |  | |  |
| 1 | Congestive heart failure | 24.42 |  | 29.16 | | 24.42 | |  | -0.11 | | | 0.00 | |  |
| 2 | Cardiac arrhythmias | 19.47 |  | 22.70 | | 19.47 | |  | -0.08 | | | 0.00 | |  |
| 3 | Valvular disease | 7.26 |  | 9.95 | | 7.26 | |  | -0.10 | | | 0.00 | |  |
| 4 | Pulmonary circulation disorders | 9.13 |  | 8.86 | | 9.13 | |  | 0.00 | | | 0.00 | |  |
| 5 | Peripheral vascular disorders | 21.12 |  | 19.98 | | 21.12 | |  | 0.02 | | | 0.00 | |  |
| 6 | Hypertension uncomplicated | 72.72 |  | 73.90 | | 72.70 | |  | -0.02 | | | 0.00 | |  |
| 7 | Hypertension complicated | 17.93 |  | 20.20 | | 17.93 | |  | -0.05 | | | 0.00 | |  |
| 8 | Paralysis | 2.64 |  | 2.89 | | 2.64 | |  | -0.01 | | | 0.00 | |  |
| 9 | Other neurological disorders | 3.85 |  | 3.22 | | 3.85 | |  | 0.03 | | | 0.00 | |  |
| 10 | Chronic pulmonary disease | 29.37 |  | 30.84 | | 29.37 | |  | -0.03 | | | 0.00 | |  |
| 11 | Diabetes uncomplicated | 12.54 |  | 13.13 | | 12.54 | |  | -0.01 | | | 0.00 | |  |
| 12 | Diabetes complicated | 20.24 |  | 18.87 | | 20.24 | |  | 0.03 | | | 0.00 | |  |
| 13 | Hypothyroidism | 11.44 |  | 18.14 | | 11.44 | |  | -0.21 | | | 0.00 | |  |
| 14 | Renal failure | 20.79 |  | 18.92 | | 20.79 | |  | 0.04 | | | 0.00 | |  |
| 15 | Liver disease | 3.19 |  | 1.98 | | 3.19 | |  | 0.07 | | | 0.00 | |  |
| 16 | Peptic ulcer disease excluding bleeding | 24.42 |  | 29.16 | | 24.42 | |  | -0.11 | | | 0.00 | |  |
| 17 | *Dropped due to collinearity (matched exclusion criteria)* | | | | | | | | | | | | | |
| 18 |  |  |  |  |  |  |  |  |  |  |  |  |  |  |
| 19 |  |  |  |  |  |  |  |  |  |  |  |  |  |  |
| 20 |  |  |  |  |  |  |  |  |  |  |  |  |  |  |
| 21 | Rheumatoid arthritis | 6.05 |  | | 6.75 | | 6.05 | | |  | -0.03 | | 0.00 | |
| 22 | Coagulopathy | 30.47 |  | | 24.84 | | 30.47 | | |  | 0.12 | | 0.00 | |
| 23 | Obesity | 5.50 |  | | 4.89 | | 5.50 | | |  | 0.03 | | 0.00 | |
| 24 | Weight loss | 28.49 |  | | 32.80 | | 28.49 | | |  | -0.10 | | 0.00 | |
| 25 | Fluid and electrolyte disorders | 1.21 |  | | 0.62 | | 1.21 | | |  | 0.05 | | 0.00 | |
| 26 | Blood loss anemia | 4.29 |  | | 4.86 | | 4.29 | | |  | -0.02 | | 0.00 | |
| 27 | Deficiency anemias | 11.44 |  | | 7.94 | | 11.44 | | |  | 0.11 | | 0.00 | |
| 28 | Alcohol abuse | 1.76 |  | | 1.27 | | 1.76 | | |  | 0.04 | | 0.00 | |
| 29 | Drug abuse | 1.98 |  | | 2.27 | | 1.98 | | |  | -0.02 | | 0.00 | |
| 30 | Psychoses | 34.65 |  | | 30.07 | | 34.65 | | |  | 0.10 | | 0.00 | |
| 31 | Depression | 6.05 |  | | 6.75 | | 6.05 | | |  | -0.03 | | 0.00 | |
| **Pharmacy-based groups** | |  |  | |  | |  | | |  |  | |  | |
| 1 | Antiplatelet | 7.70 |  | | 7.16 | | 7.70 | | |  | 0.0204 | | 0.00 | |
| 2 | Anticoagulant | 11.00 |  | | 12.69 | | 11.00 | | |  | -0.0541 | | 0.00 | |
| 3 | Epilepsy | 9.79 |  | | 8.41 | | 9.79 | | |  | 0.0463 | | 0.00 | |
| 4 | Hypertension | 17.71 |  | | 17.55 | | 17.71 | | |  | 0.0042 | | 0.00 | |
| 5 | HIV | 0.33 |  | | 0.10 | | 0.33 | | |  | 0.0399 | | 0.00 | |
| 6 | Tuberculosis | 0.55 |  | | 0.29 | | 0.55 | | |  | 0.0353 | | 0.00 | |
| 7 | Rheumatic conditions | 64.47 |  | | 51.31 | | 64.45 | | |  | 0.2748 | | 0.00 | |
| 8 | Hyperlipidemia | 31.90 |  | | 31.34 | | 31.90 | | |  | 0.0120 | | 0.00 | |
| 9 | Malignancies | 0.11 |  | | 0.17 | | 0.11 | | |  | -0.0191 | | 0.00 | |
| 10 | Parkinson’s disease | 2.75 |  | | 2.21 | | 2.75 | | |  | 0.0329 | | 0.00 | |
| 11 | Renal disease | 0.66 |  | | 0.42 | | 0.66 | | |  | 0.0297 | | 0.00 | |
| 12 | End stage renal disease (ESRD) | 0.22 |  | | 0.10 | | 0.22 | | |  | 0.0254 | | 0.00 | |
| 13 | Anti-arrhythmic | 2.31 |  | | 4.47 | | 2.31 | | |  | -0.1435 | | 0.00 | |
| 14 | Ischemic heart disease/Angina | 6.60 |  | | 7.65 | | 6.60 | | |  | -0.0422 | | 0.00 | |
| 15 | Congestive heart failure | 64.36 |  | | 68.01 | | 64.34 | | |  | -0.0762 | | 0.00 | |
| 16 | Diabetes | 19.36 |  | | 19.71 | | 19.36 | | |  | -0.0087 | | 0.00 | |
| 17 | Glaucoma | 4.18 |  | | 4.37 | | 4.18 | | |  | -0.0093 | | 0.00 | |
| 18 | Liver failure | 0.22 |  | | 0.68 | | 0.22 | | |  | -0.0980 | | 0.00 | |
| 19 | Acid peptic disease | 60.95 |  | | 56.85 | | 60.93 | | |  | 0.0840 | | 0.00 | |
| 20 | Transplantation | 0.66 |  | | 0.65 | | 0.66 | | |  | 0.0012 | | 0.00 | |
| 21 | Respiratory illness, asthma | 98.46 |  | | 88.77 | | 98.46 | | |  | 0.7869 | | 0.00 | |
| 22 | Thyroid disorders | 23.10 |  | | 20.25 | | 23.10 | | |  | 0.0675 | | 0.00 | |
| 23 | Gout | 14.19 |  | | 14.30 | | 14.19 | | |  | -0.0031 | | 0.00 | |
| 24 | Inflammatory bowel disease, chronic | 1.65 |  | | 0.85 | | 1.65 | | |  | 0.0626 | | 0.00 | |
| 25 | Pain and inflammation | 62.16 |  | | 56.28 | | 62.14 | | |  | 0.1211 | | 0.00 | |
| 26 | Pain | 18.15 |  | | 16.21 | | 18.15 | | |  | 0.0505 | | 0.00 | |
| 27 | Depression | 29.70 |  | | 24.65 | | 29.70 | | |  | 0.1106 | | 0.00 | |
| 28 | Psychotic illness | 5.61 |  | | 4.76 | | 5.61 | | |  | 0.0371 | | 0.00 | |
| 29 | Bipolar disorders | 0.22 |  | | 0.25 | | 0.22 | | |  | -0.0055 | | 0.00 | |
| 30 | Anxiety and tension | 11.88 |  | | 10.13 | | 11.88 | | |  | 0.0540 | | 0.00 | |
| 31 | Hepatitis | *Dropped due to collinearity* | | | | | | | | | | | | |
| 32 | Ischemic heart disease | 48.18 |  | | 52.39 | | 48.18 | | |  | -0.08 | | 0.00 | |
| TM = Telemonitoring | | | | | | | | | | | | | | |
| SMD = Standardized mean difference | | | | | | | | | | | | | | |
